# Trial for Mal-practice

**Published:** 1849

**Authors:** 


### ARTICLE VII.

### TRIAL FOR MAL-PRACTICE.

KENDALL CIRCUIT COURT.

Judah Rowley, *vs.* Charles H. Duck, and Joseph Morley.

Before the Hon. T. L. DICKEY, Circuit Judge.

This was an action on the case, against Defendants as Surgeons, for mal-practice. The charges in the declaration were for "unnecessarily, unskillfully and with a *carpenter dull saw*, cutting off Plaintiff's leg." It also alledged *negligence* in the treatment. *Defendants plead not guilty.*

Messrs. Dodge & Jones, Attorneys for Plaintiff; J. N. Arnold, for Duck; Helm, for Morley.

The following is, in substance, a brief statement of the case as made by Jones, in his opening on the part of the plaintiff:—

During the month of August, 1844, Rowley, the plaintiff, received a severe injury of the foot from a thrashing machine. Dr. Morley, one of the defendants, after examining the injured limb soon after the accident, decided that the extent o the injury was such as to require amputation of the leg, and accordingly sent for the other defendant, Dr. Duck, who was then in the neighborhood on a visit, and requested him to perform the operation who, as we contend, without careful examination or consultation with the other physicians present, proceeded at once to operate, and that, too, with improper instruments, and at a point near the knee, too high up upon the leg. We expect to show in evidence, that the injury did not extend above the instep and that, therefore, if an operation was required, it should have been performed upon the foot so as to leave all or a part at least, of the bones of the tarsus.

We propose to show also, that the amputation, if necessary, should have been performed near the ankle joint; also, that as the effect of want of skill in operating and negligence in the subsequent treatment, the patient has a bad stump, from which he suffers much pain and inconvenience.

Arnold, on the part of defendants, stated on the other hand, in substance as follows:—

We expect to show in evidence, that the injury extended into, and even above the ankle joint, that it was of such a nature as to require amputation of the leg, that the proper point was the one selected, and that there was no want of judgment or skill in operating, or lack of attention subsequently, on the part of defendants; also, that the plaintiff, so far from suffering pain and inconvenience, is remarkably healthy, active, and free from pain.

The witnesses on the part of the plaintiff were now called and sworn.

James P. Lamb testified in substance as follows:.--

Was present at the time of operation. Dr. Morley arrived soon after the accident, and after making a slight examination of the injured limb, went out for Dr. Duck. During his absence, Dr's. Seely and Holden arrived. After the return of Dr. Morley with Dr. Duck, a short consultation was held by the physicians present, which resulted in the determination to amputate.

The operation was performed by Dr. Duck. Time occupied about half an hour. Was a long time in sawing off the bone. Instruments used, a long knife and carpenter saw. From the appearance of the injured limb, both before and after amputation, believes the injury did not extend so as to effect the bones higher than the instep.

Henry Ford called.

Was present at time of injury and also during the operation. Foot appeared badly injured as high as instep, (the witness here marked out upon his foot the point to which the injury appeared to him to extend,) Dr. Duck operated. Instruments used, a handkerchief twisted around the thigh to prevent bleeding, a long knife and a saw, obtained from a carpenter's shop near by. Time occupied in sawing off bone, fifteen or twenty minutes. Saw worked badly.

Cross-examined.---Dr's. Duck, Morley, Holden and Seely were all present and took part in the consultation and operation. Dr. Duck remarked that his amputating instruments were in Chicago, (forty miles distant,) and that as the weather was warm it would be unsafe to delay the operation until they could be sent for.

Mr. Bremer called.

Saw the stump two or three days after amputation, at which time, Dr. Morley extracted from the wound a flat piece of bone two or three inches in length. Dr. Morley said at the time he thought it probable that this piece of bone had been broken off by the saw, at the time of the amputation. Dr. Morley stated also, in a subsequent conversation with witness, that, in his opinion, the leg ought not to have been amputated, but that he consented that it should be taken off to gratify Dr. Duck, who wished to operate.

Cross-sxamined.—The piece of bone came out two or three weeks after amputation, plaintiff then setting up, and in four or five weeks after the operation was up and about. Conversation with Dr. Morley, concerning the operation, took place some time after operation. He sometimes drinks to excess, might have been excited when he made the remark.

Lewis Sutton called.

Was present at the time of operation. Testimony, in substance, same as that of the two first witnesses.

George Hay, tailor, called.

Plaintiff has resided with and worked for witness, for three or four years past. He suffers no inconvenience or pain from working in shop as tailor, but the stump becomes sore, blisters, and is painful after severe exercise.

Cross-examined.—Plaintiff walks as fast as any other man, short distances. Is a leader in sports. Has no property.

D. Johnson called.

Witness is a carpenter. Lent the saw, used in amputating the leg, to Dr. Morley. Saw not in very good condition. Dr.

Morley said it would answer. It was a dove-tail saw, not set to saw across the grain.

Dr. Hopkins called.

Is a practicing physician and surgeon. Has heard all the testimony, but from the evidence given, can neither form an opinion, with regard to the nature of the injury, or decide as to what would have been the proper course for a surgeon to pursue in the case.

Dr. Hard called.

Is a practicing physician and surgeon at Aurora, also a professor in the Indiana Medical College. Has heard the testimony. Believes, taking the statements of witnesses as the basis upon which to form an opinion, that by performing Chopart's operation, a portion of the foot might have been saved. Has examined the stump, and finds it a tolerably good one. It is a little too short and would have been better if the flap had adhered, so as to serve as a cushion for the bone.* Not material as to what instruments are used in amputating, providing they answer the purpose. From evidence it appears that defendants were a longer time in amputating than is generally required to perform such an operation, but they are not to be considered culpable on that account, as it should be a rule in surgery to operate skillfully rather than rapidly.

Cross-examined.—Would not be willing to give a professional opinion as to what operation should have been performed, based upon the testimony now before the court. Should much prefer the judgment of physicians. Opinion based upon the assumption that neither the tibia, fibula, joint, or large bones of the tarsus were seriously injured.

In cases where the ankle joint is laid open, and where the tendons and ligaments are lacerated and torn amputation is

*The bone appeared as if it had been divided about an inch below the tuberkle of the tibia, and the mnscles had retracted so as to form a prominence above and behind it.

justifiable some, in cases necessary. Lacerated wounds are more dangerous than incesed, and injuries of the leg or foot more serious than those of the arm or hand.

The sawing of a bone causes but little if any pain. Believes that a bone might be split in sawing, just before the section is completed, especially if much motion be permitted.

Dr. D. Eastman called.

Is a practicing physician and surgeon. Considers the testimony as to the nature and extent of the injury too imperfect to justify an expression of opinion, as to what should have been the treatment in the case in question. In cases where the phalangeal, metatarsal, and even where the small anterior tarsal bones are included in an injury, an attempt should be made in most instances to save a portion of the foot, but if on the other hand, an injury is so extensive as to seriously effect the ankle joint amputation is justifiable. Should put more confidence in the opinion of physicians in attendance at the time of an operation than upon the testimony of all others present.

At this stage of the proceedings a discussion arose between the parties in consequence of an attempt made on the part of the plaintiff to introduce testimony to show what are the pecuniary circumstances of the respective parties. By mutual agreement it was finally determined to wave the subject. No decision was made by the court, therefore, as to whether such testimony is admissable or not in such cases.

The witnesses on the part of defendants now came forward and were sworn.

Mr. Ives, upon being called.

Is acquainted with both parties. Has known plaintiff five or six years. Was present at the time of operation. From appearance of injured limb, previous to amputation, believes the parts were lacerated and torn around, and even above, the ankle joint upon its internal side. Dr. Seely was the only physician present at the time witness arrived at Lamb's house, where the operation was performed.

Drs. Duck and Morley came together, and in a short time Dr. Holden arrived.

All four of the physicans above named took part in the examination of the injured limb. Dr. Duck was particular in examining below, around, and above the ankle joint. He then stated that the joint was injured.

A consultation was then held, which resulted, as witness believes, in the unanimous conclusion of the physicians present, that the injury was so severe and extensive as to require amputation of the leg above the ankle joint. The bones and soft parts torn from the limb, were taken from the machine by witness. Some of the bones were long with a rounded head; others broad and thick, not as long. Some in fragments others apparently entire. Time occupied in performing operation believes to have been 10 or 15 minutes.

Cross-examined.—The injury about the instep appeared as if it had been produced by a tooth of the machine passing through the foot near to or at the ankle joint. (Testimony the same in substance as before given.)

Dr. Seely called.

Is a practicing physician and surgeon. Was present and took part in the consultation and operation. Examined the parts previous to the operation. Found the ligaments and tendons about the ankle joint lacerated and broken, and some of the small bones of the tarsus torn from their sockets. Foot turned downwards and inwards as in dislocations of the ankle joint. It was the opinion of all the physicians present that the injury was such as to require amputation. Doctor Duck operated. Time occupied in the operation 10 or 15 minutes.

Cross-examined.—Has been in practice since 1815, and since that time has endeavored to keep up with the improvements in surgery. Not had much practical experience, but believes himself capable of deciding correctly as to the proper treatment in such cases. Lateral ligaments of ankle joint must have been lacerated or the displacement downwards and inwards could not have taken place. Not positive as to whether capsular ligament was lacerated or not.

D. St. Clair called.

Knows plaintiff, and has conversed with him frequently concerning the injury and operation. About four weeks after the operation plaintiff told witness he was well satisfied with the treatment, and felt convinced that he should not have recovered if it had not been for the operation and attention of Dr. Morley subsequently.

Cross-examined.—Conversation with plaintiff took place about three or four weeks after operation. Plaintiff was then out and walking upon a wooden leg. Believes the plaintiff to have been about 17 years of age at the time.

—— Tolford called.

Is acquainted with plaintiff, and has conversed with him concerning the operation, &c. (Testimony same in substance as that of previous witness.)

Dr. Hopkins re-called on the part of defence.

About four years since examined the bones of amputated limb, then in possession of plaintiff. Was requested to do so by plaintiff, for the avowed purpose of witness' opinion as to whether or not, it would be advisable to bring an action against defendants for mal-practice. The small bones of the tarsus were some of them fractured. As far as memory serves, is now of opinion that both the astragulus, and oscalcis were fractured. Is positive that the tibia was fractured at the lower extremity, in such a manner as to include parts concerned in the formation of the ankle joint. The fracture of the tibia extended from the lower extremity of the bone, from one and a half to two inches upwards, upon its anterior surface. Is of opinion, taking the condition of the bones into consideration, that amputation of the leg was justifiable. Would not be considered bad practice to amputate near the knee, or at any point upon the leg which the surgeon might choose to select. Has seen the bones since the time refered to above, in the possession of plaintiff's Attorney, Jones.

Cross-examined.—Saw the bones about a year after operation. Was informed at the time that they had been buried, and were subsequently dug up. Soft parts detached at time of examination. Could not say positively whether astragulus and os calcis were injured or not, but believrd they were. Amputation just above the ankle leaves a l ng sump, to which an artificial foot may be adjusted; but believes such a stump is not as useful to a laboring man as when the amputation is made nearer the knee. The fractures of the bones in question might possibly, have been produced by accident in digging them up, or otherwise, since the operation.

Dr. Eastman re-called.

From testimony of Dr. Hopkins just given, believes that ampntation was proper in the case in question. Amputation of a portion of foot, in case of fracture of tibia, and injury of joint, as described by Dr. Hopkins would be improper. Point for amputation of leg when required may very properly be left discretionary with the surgeon. Point near the knee joint most frequently selected.

Cross-examined.—Bases opinion last given upon testimony of Dr. Hopkins.

Dr. Holden called.

Was present at the time of amputation. Examined the stump after operation. Found ends of bones smooth. The stump a good one. Time consumed in operation short.

Cross-examined.—Was a medical student at the time—not in practice. Has been in practice four years. Examined the limb amputated, and believed at the time that the operation was necessary. It was done well, and at the proper place.

Dr. Hard re-called on the part of the plaintiff.

Had a conversation with Dr. Seely soon after the operation, at which time it was stated in substance, by Dr. Seely, that in his opinion the operation was unnecessary, and the limb might have been saved.

Cross-examined.—Believes from testimony of Dr. Hopkins concerning condition of bones, that amputation near the knee joint was necessary.

Dr. Herrick called on the part of defence.

Has been a practicing physician and surgeon fourteen years. Is Professor of Anatomy in Rush Medical College, Chicago. Has served as surgeon in the war with Mexico and at the battle of Buena Vista. Believes from testimony of Dr. Hopkins, concerning condition of bones, that amputation of the leg was justifiable in the case in question. Surgeons differ as to the point to be selected in amputations of the leg. In deciding this question they are influenced and governed very much by circumstances. Indigent and laboring men are generally better off with short stumps. Has examined the stump, but finds nothing to object to in its condition or appearance. Believes, from testimony of Dr. Hopkins, that Shoupart's operation should not have been performed in the case of Plaintiff.

The testimony being now closed, it was proposed by the council for defendants to submit the case to the jury without arguments. This, however, was not agreed to by the opposite council. The arguments on both sides were creditable. The gentlemen on the part of plainiiff, being led astray, as we svppose, by their zeal in behalf of their client, stated that the testimony of medical men could not fully be relied on in such cases, as they would generally sustain each other, right or wrong. This is certainly a most unjust and uncalled for accusation, since no class in the community are more anxious to discourage quackery and punish ignorance, under whatever garb it may appear, than the well-informed medical men, constituting the great body of the profession.

The arguments on the part of the defence were very properly, as we think, based on facts in the evidence given by medical men, and upon the assumption that they constitute the proper authority to which we must look for correct, and just decisions in such cases.

The following are the principal and most important instructions given to the jury by the Court.

If the Jury believe from the evidence, that the defendants were by profession physicians and surgeons, and in such capacity undertook to operate upon, dress or heal the plaintiff's wounded foot or leg, and did unskilfully operate upon the same ; or did unskilfully, or negligently dress or take care of the same after performing said operation, so that the Plaintiff sustains any damage by reason of such unskillfulness or negligence of said defendants, the law is for the Plaintiff.

If the Jury find for the Plaintiff, it will be their duty to allow damages to the Plaintiff, commensurate with the injury which they believe he has sustained, by reason of the negligence or unskilfullness of Defendants, in all not exceeding the amount ($5,000) claimed in Plaintiff's declaration.

The Plaintiff in this case must establish affirmatively, that the Defendants have been guilty of negligence or mal-practice, in order to justify them in finding a verdict for Plaintiff.

If the Jury believe that the defendants acted, in good faith, with ordinary skill as surgeons and in pursuance of the usual practice, then they should not find the defendants guilty.

If the Jury believe from the evidence that there is a difference of opinion among surgeons in regard to the proper treatment, and that there are good authorities both ways, so as to leave it an unsettled question among good surgeons, then if they believe from the evidence that the defendants acted according to their best judgment, and are sustained by good authorities, and not in revelation of any well-settled rule of the profession, then the Plaintiff is not entitled to recover. Unless the Jury believe there was a joint wrong in this case by both defendants, the Plaintiff cannot recover against both of the Defendants.

That the admissions of Morley furnish no evidence against Duck, and that it is the duty of the Jury to disregard them entirely so far as Dr. Duck is concerned. If the Jury believe from the evidence, that the amputation was made at the place most usually made by the best surgeons, and in pursuance of established authorities, then although there may be authorities in favor of operating at another place, still the fact of amputation near the knee is not, by reason thereof, mal-practice.

If the believe Jury believe from the evidence, that Morley stated after the amputation that it was improperly done, such statement is not conclusive as to him; but it is the duty of the Jury to take into consideration the evidence of the case tending to shew the statement not to be correct.

The verdict of the Jury was, no cause of action. H.

